# Flavor Dependent Retention of Remote Food Preference Memory

**DOI:** 10.3389/fnbeh.2017.00007

**Published:** 2017-02-02

**Authors:** Aditya Singh, Suraj Kumar, Vikram Pal Singh, Asish Das, J. Balaji

**Affiliations:** Centre for Neuroscience, Indian Institute of ScienceBangalore, India

**Keywords:** remote memory, social transmission of food preference (STFP), mouse behavior, sensitivity, performance, flavor, innate preference, simulation

## Abstract

Social Transmission of Food Preference (STFP) is a single trial non-aversive learning task that is used for testing non-spatial memory. This task relies on an accurate estimate of a change in food preference of the animals following social demonstration of a novel flavor. Conventionally this is done by providing two flavors of powdered food and later estimating the amount of food consumed for each of these flavors in a defined period of time. This is achieved through a careful measurement of leftover food for each of these flavors. However, in mice, only a small (~1 g) amount of food is consumed making the weight estimates error prone and thereby limiting the sensitivity of the paradigm. Using multiplexed video tracking, we show that the pattern of consumption can be used as a reliable reporter of memory retention in this task. In our current study, we use this as a measure and show that the preference for the demonstrated flavor significantly increases following demonstration and the retention of this change in preference during remote testing is flavor specific. Further, we report a modified experimental design for performing STFP that allows testing of change in preference among two flavors simultaneously. Using this paradigm, we show that during remote testing for thyme and basil demonstrated flavors, only basil demonstrated mice retain the change in preference while thyme demonstrated mice do not.

## Introduction

Memories that can be retrieved long after acquisition are termed as remote memories (Frankland and Bontempi, 2005; Squire and Bayley, 2007). According to the standard view of systems consolidation (SC), memory encoding is hippocampus-dependent, and the retrieval of memories becomes less dependent on hippocampus with time (Kim and Fanselow, 1992; Squire et al., 2015). Differential retrieval of details in recent and remote memories is one of the intensely researched areas in the field of learning and memory (Anagnostaras et al., 1999; Frankland et al., 2004; Teixeira et al., 2006; Restivo et al., 2009; Goshen et al., 2011; Tayler et al., 2013; Zovkic et al., 2014; Barry et al., 2016). Researchers have adopted various approaches to understand the underpinnings of recent and remote memory through different behavioral paradigms. One of these approaches, social transmission of food preference (STFP), provides us with a unique single-trial training paradigm for studying the retrieval of non-aversive, hippocampal dependent memory (Countryman et al., 2005; Ross and Eichenbaum, 2006; Smith et al., 2007). Further, STFP allows one to probe how SC of declarative memory occurs relatively independent of spatial navigation.

STFP was developed by Bennet G Galef Jr. during 1970's with rats and since then, few modifications have been incorporated to implement this learning paradigm in mice (Galef, 1977; Wrenn et al., 2003; Choleris et al., 2011). Social animals such as rodents use multimodal sensory information in the form of olfactory, tactile, vocal, and visual cues to identify and share information with foragers. In case of mice, information for palatable food is usually shared between forager (demonstrator mice or DemoMice in our case) and observer (ObMice in our case) at a location distant from feeding site (Galef, 1977). ObMice develop a preference for a flavor when it is detected along with the breath of DemoMice. Specific odor components arising from the successful digestion of consumed food i.e., carbon disulphide or carbonyl sulfide (Galef et al., 1988) help establish an association between consumed flavor and its safety (Choleris et al., 2009). After a conspecific eats and stays healthy or breathes after ingesting novel flavor being demonstrated, the ObMice learn about this flavor being safe for consumption. Later, if ObMice are presented with two flavors simultaneously, one entirely novel and another already demonstrated and detected along with specific breath components of DemoMice, the ObMice consume more of demonstrated flavor (demoFlavor).

For quantifying the retained associations formed during STFP, usually, weight of food containers is recorded before and after the sessions to calculate “weight of consumed food” (W_C.F_). In order to avoid toppling of food containers by experimental mouse, these containers are heavy and their weight is in the range of ~100 g. During STFP testing sessions, the mice typically consume ~1 g of flavored food. However, in such studies spillage of food by itself can be quite substantial. Correcting for such errors on top of measuring a small difference in weight of the food containers makes the measurement tedious and hence the estimation of food consumption during STFP error-prone while performing these studies in mice. As a result, it becomes difficult to detect small changes associated with the strength and nature of the memory. One alternative could be video tracking and estimating the food consumed through the measurement of time spent near the food containers. Previous efforts to correlate time spent around the food containers with consumed food in STFP paradigm were instrument intensive (Plucinska et al., 2012) and did not provide analytical measures to elucidate the performance. Alternatively, we propose and utilize the “number of food consumption episodes” obtained through video analysis as a measure of performance. Using this method, we establish a STFP procedure that is easy to implement and more sensitive.

## Materials and methods

**Figure 1:**
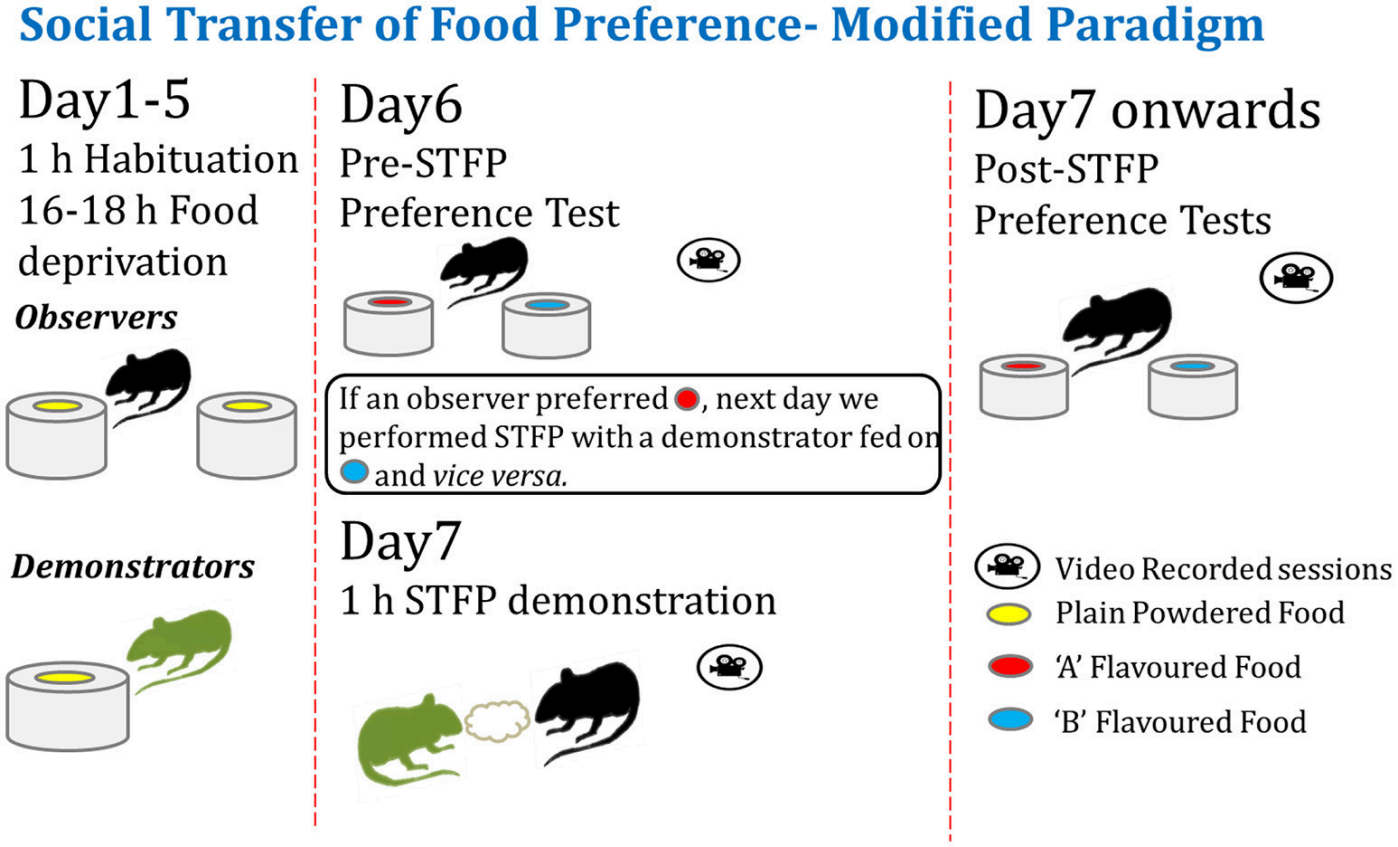
**Modified experimental paradigm for social transmission of food preference behavior**. **Left**—Observer and demonstrator mice are singly housed in individually-ventilated cages and habituated for a daily food deprivation of 16–18 h. Observers are habituated to eat from two cups of plain powdered chow kept at two ends of experimental cage. Demonstrators are habituated with one cup of plain powdered chow in their home cage (left panel). **Center**—After ~4–5 days of habituation, pre-STFP preference test is performed by providing two novel flavors in two cups. Next day, less preferred flavor is fed to respective DemoMice for an hour and they are immediately released in experimental cage with ObMice for 1 h of STFP session. **Right**—~24 h later, ObMice are tested for post-STFP preference of novel flavors. Testing and demonstration sessions are video monitored.

As depicted in Figure 1, observer and demonstrator animals were habituated for food deprivation and consumption during the training and testing sessions. We conducted a pre-STFP preference test with experimental animals in order to identify the flavor preferences for each animal. Then the animals were segregated in two groups based on their flavor preferences. These groups were then demonstrated the non-preferred flavor during the social interaction as described below.

### STFP paradigm

C57/B6 mice (~2 months old) were housed in 12 h light/dark cycle. All the experiments were conducted during the light-on phase (06.30–18.30 h). The demonstrator and ObMice were single housed in individually ventilated cages (IVC; 36 cm long × 14 cm wide × 12.5 cm high; Citizen, India). For testing the performance pre- and post-STFP, fresh cages similar in size and shape to their home cages were used as experimental cages. These cages contained spill-proof aluminum trays designed to fit in experimental cage. Customized cylindrical plastic food containers of same dimensions were fixed at the center of these trays and kept at two opposite ends of the testing chamber (**Figure 4C**). The bottom portion of food containers was filled with bedding material, leaving sufficient space to keep a vessel filled with flavored food. A hole of ~2 cm diameter was made in the lid to provide access to flavored food provided in closed food cups. In order to allow video recording from top-view, the cages were covered with transparent rectangular perspex sheets, with holes for ventilation along the short central midline. Also, since the arrangement of food containers in test cage was symmetrical along longitudinal axis (**Figure 4C**), pseudo-randomization of containers was achieved by reversing the order of container placement. The testing chambers were arranged in 3 × 4 or 4 × 4 configurations, as required, on the floor of the room in which animals were housed for the experiment. Semi-transparent walls of testing chambers were further blocked by placing cardboards outside all chambers to avoid the visual distractions for mice. A HD webcam (Model c920, Logitech) was mounted on top (~4 ft above floor) for recording movements from all the experimental cages in field of view to obtain high resolution images.

Animals were handled for a week (daily for ~10 min) before habituation. Both demonstrator and ObMice were habituated for ~15 h of daily food deprivation to remove any effect of surprise associated with sudden food deprivation affecting the performance. Habituations were performed until ObMice ate equally from both cups without any spatial-preference during 1 h of experimentation session. Typically this was achieved in ~ 5–7 days. Food deprivation was continued from the beginning of habituation sessions until the post-STFP preference test conducted after 24 h. For 17- and 41 day remote preference tests, ~15 h deprivation was started 3 days in advance before the testing day. Water was provided *ad libitum* except for 1 h of experimentation/habituation session. A food pellet (Batch#0001904678, Nutrilab Rodent Feed) of 1 g was provided after each session in the home cage to fulfill the daily nutritional requirements.

DemoMice were habituated for 1 week to eat regular food pellets in powdered form provided in the customized food container in their home cage. ObMice were habituated in test chambers to eat powdered food pellets from two cups fixed to aluminum trays (Figures 1, **4C**). After the habituation, pre-STFP preference test was conducted by presenting the ObMice with two food cups for 1 h in the testing chamber. In this study we used two flavor pairs, (i) cocoa and cinnamon and (ii) thyme and basil. Cocoa-cinnamon group was presented with 2.0% cocoa- and 1.0% cinnamon- flavored powdered food while thyme-basil group was presented with 1.0% thyme- and 0.8% basil flavored powdered food. Flavoring agents were acquired as commercially available condiments (SNAPIN herbs and spices, Lotus household product, India). Flavor concentrations were chosen based on previous studies (Holmes et al., 2002; Ross and Eichenbaum, 2006; Smith et al., 2007; Lesburguères et al., 2011) and from pilot experiments conducted in our lab.

Food cups were weighed before and after the sessions. Aluminum trays were weighed following each session to account for spilled food. Using the data from pre-STFP preference test, we identified the lesser consumed flavor as less preferred flavor (flavor with <0.5 preference in all cases except for one animal in cocoa-cinnamon experiment with 0.57 preference and one animal in thyme-basil experiment with 0.53 preference) for that mouse. Later for demonstration session, plain powdered chow mixed with less preferred flavor was given to respective DemoMice in their home cage for an hour. Following this, DemoMice were immediately transferred to the home cage of ObMice for 1 h to interact and demonstrate the initially less preferred flavor. Flavor demonstration was conducted 1 day after pre-STFP test for cocoa-cinnamon experiment and 5 day after pre-STFP test for thyme vs. basil experiments. Post-STFP preference test was conducted for ObMice in a similar manner as of pre-STFP test. All the testing sessions were video monitored and recorded. Freshly flavored food was prepared every day before the experiment.

In a separate experiment using a different group of mice (*n* = 10), we determined the stability of the preference exhibited during pre-STFP. For this experiment, animals were habituated in same manner as previous experiment. After conducting first test on day 1, animals were tested a second time without intervening demonstration of any flavor. We tested the preference for three flavor pairs (i) Cocoa 2%—Cinnamon 1% (the pair used in experiment) (ii) Cumin 0.5%—Dry Mango 1% and (iii) Basil 0.7%—Mint 1%, at the intervals of 14, 4, and 1 days, respectively. All the behavior experiments were approved by animal ethics committee of Indian Institute of Science, Bangalore, India.

### Image analysis and automation

Open source software ImageJ was used to process the videos for generating heat maps. Background for each 1 h long video was generated by averaging intensities across all the frames of the video recorded @ 30 fps (**Figure 4C-top**). Background for each cage was then subtracted from image stack of respective cages. Video processing was conducted with in-built functions in ImageJ software using maximum entropy threshold and analyze particle. This allowed us to set threshold parameters and particle size ROI for visualizing only the mouse body in the cage (**Figure 4B**). Heat maps were then generated by summation of all the processed frames of individual cage stacks (**Figure 4A**).

### Scoring of eating episodes

One hour videos for all the testing sessions were analyzed to record the position of each mouse in either left or right half of the cage. Each cage was continuously observed and mice positions were noted after every 10 s (300 frames). In this manner, each video of 3600 s was scored to get 360 data points. Animal visits to a region of interest were accounted as an “eating episode” only if they stayed for 30 s (900 frames) or more continuously at a stretch in that region. Position of mice was marked to be in the center of the cage when it could not be assigned to left or right half of the cage. Episodes with center mice position were not accounted for calculating preferences. Scorer was blind to the information about flavor in food cups. **Figures 5**–**8** were generated using eating episode data. This scoring was independent of the video processing for heat map generation.

### Data analyses

#### Calculation of preferences

In order to quantitatively express the demoFlavor preference w.r.t either weight or time as a measure, we define mean preference as follows:

$$\begin{array}{l} Mean\text{Preference}\langle {\text{P}}^{\text{DEM}}\rangle =\langle \frac{{\text{W}}^{\text{DEM}}}{{\text{W}}^{\text{DEM}}+{\text{W}}^{\text{NON}-\text{DEM}}}\rangle or\\ \;\langle \frac{{\text{T}}^{\text{DEM}}}{{\text{T}}^{\text{DEM}}{+\text{T}}^{\text{NON}-\text{DEM}}}\rangle \end{array}$$

where, W^Dem^ and W^Non-Dem^ = Consumed weight of demoFlavor and non-demoFlavor, respectively; T^Dem^ and T^Non-Dem^ = Time spent near demoFlavor and non-demoFlavor container, respectively.

#### Statistical analyses

Mean comparison analyses for total weight and time measurements were performed using one way, repeated measure ANOVA with Mauchy's test for validating the assumption of sphericity across datasets. Sphericity violation found while comparing the W_C.F_ for thyme DemoMice across successive testing sessions was compensated by performing Greenhouse-Geisser correction. Pearson's *r* was used to ascertain the existence of correlation.

#### Curve fitting

In order to estimate the amount of time spent eating a flavor, we used the following procedure. For a given group of mice, we arrived at an average consumption profile by estimating the cumulative eating episodes as a function of time. These consumption profiles show the variation in the “rate of consumption” for a given flavor across the duration of a testing session. We compared such cumulative time-based consumption profiles for each testing session after fitting them to the integral of Weibull function (Fox and Byerly, 2004). Weibull cumulative distribution function (Weibull CDF) used for fitting is as follows:

$$\begin{array}{l} y=A\cdot \left(1-e^{-{\left(k\cdot x\right)}^{d}}\right)+b \end{array}$$

Parameter *A* is the amplitude of the fit representing the cumulative consumption during the session; parameter *k* is the slope representing the decline in rate of consumption; parameter *d* represents the deviation of the fit from exponential indicating the duration for which initial intake rate is maintained; and parameter *b* is the offset to the fit. For the cases where the fit with a freely varying *b* parameter did not converge, *b* was fixed at zero (For demoFlavor consumption profiles: 17- and 41 days post-STFP for both thyme and basil; Non-demoFlavor consumption profile: 24 h post-STFP for thyme). In the scenario when parameter *k* was close to zero and parameter *d* was close to 1, the Weibull function was best approximated to a straight line. For such cases in our data, CTPs were best fit to a straight line. Amplitude was calculated from the slope as a product of slope and total time of the session in minutes. (This was conducted for following demoFlavor consumption profiles: pre-STFP CTPs for both thyme and basil; Non-demoFlavor CTPs: pre-STFP, 24 h and 17 days post-STFP for Basil).

These amplitudes were compared by conducting one way ANOVA using mean and standard error of mean obtained from fits (**Figure 8**). Degrees of freedom (d*f*) for such comparisons were arrived at by using the following Equation:

$$\begin{array}{l} df=\left(\#\text{datapoints}\right)-\left(\#\;\text{free parameters used in the fit}\right) \end{array}$$

Curve fitting was performed using Origin Pro (ver. 8.5) software.

### Animal exclusions

In order to capture the maximum possible behavioral variations in the populations, no animals were excluded from the analyses.

## Results

### Preference estimation through weight measurement

Previous experimental design for performing STFP required the use of a separate group of mice to establish baseline preference for a given flavor pair. In these designs, the flavor pairs are chosen with an assumption that both the flavors are preferred equally on an average. Such a design would be fine if the distribution of the preference in a group is homogeneous and is equal for both the flavors. However, that is often not the case and usually there are sub-groups of mice that prefer one flavor more than the other. These preferences could be innate and thereby remain invariant over time. In such a scenario, conventional design of random assignment of flavor to be demonstrated could result in demonstration of an already preferred flavor to a substantial fraction of mice. This reduces the power of the experiment to detect and measure changes due to demonstration, especially when the innate preference (IP) contribution is high (“ceiling effect”). Such designs lack the ability to counter-balance the flavors according to innate preference of experimental mice. Our method as illustrated in Figure 1 overcomes this issue. In our design, we determine pre-STFP preference for each mouse and hypothesize that this has a major contribution from IP. The less preferred flavor is chosen to be the demoFlavor for an individual mouse. This allows us to conduct demonstration in a counter balanced manner. Using this new approach, we wanted to establish that post-STFP change in preference for demoFlavor is a measure of STFP memory as observed in conventional protocols for STFP.

#### Establishing modified STFP procedure using cocoa-cinnamon flavor pair

**Figure 2:**
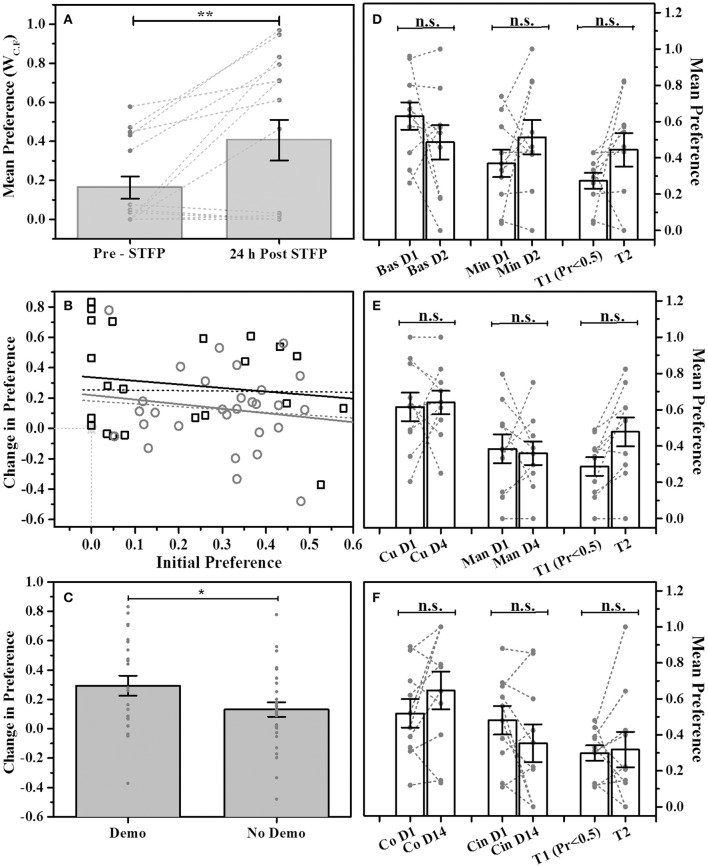
**STFP mediated change in preference. (A)** Preference comparison using weight of consumed food (W_C.F_) for demonstrated flavor—cocoa—vs. cinnamon -flavored food. Mice were presented with cocoa and cinnamon flavored food for preference test conducted before and after STFP (24 h pre- and post-STFP; *n* = 15). Each bar represents mean flavor preference for 15 mice. 3 out of 15 mice showed zero preference for both the tests and hence their data points are overlapping at zero. Means were calculated using two approaches (i) by including the animals with no consumption during both the tests (*n* = 15) and, (ii) by excluding the animals with no consumption during both tests (*n* = 12). Significance was calculated using paired-sample *t*-test at 0.05 level of significance. For *n* = 15, 24 h post-STFP, preference for demoFlavor (preference = 0.41 ± 0.10) was significantly more than that for pre-STFP (preference = 0.16 ± 0.06; *t*-Statistic = −3.11, *p* > |t| = 0.008). For *n* = 12, we observe an increased significance (pre-STFP preference = 0.20 ± 0.07, 24 h post-STFP preference = 0.51 ± 0.11; paired-sample *t*-test: *t*-Statistic = −3.39, *p* > |t| = 0.006; plot not shown). **(B)** Correlation between initial preference (<0.5) measured during first preference test and the change in preference measured during second test for animals with STFP (black hollow squares; *n* = 23 black solid line and *n* = 26 black dashed line) and without STFP (gray hollow circles; *n* = 29 gray solid line and *n* = 30 gray dashed line). Correlation for both the animal groups was low indicating that change in preference does not depend on initial preference. Higher intercept for STFP group indicates effect of STFP on change in preference. Dashed lines represent correlation for all the animals of respective groups. Solid lines represent correlation for animals with non-zero initial preference and non-zero change in preference for respective groups. Animals with zero preference (3 mice for STFP group and 1 mouse for no-STFP group) are represented by overlapping hollow star at zero intersection of x- and y -axis. **(C)** Comparison of change in preference for demonstrated (Demo) vs. non-demonstrated (No Demo) animals. Average change in preference for demonstrated mice (0.294 ± 0.067; *n* = 23; measured across one test session before demonstration and another session just after demonstration) was higher than that for non-demonstrated animals (0.131 ± 0.049; *n* = 29; tested across two tests without demonstration using data as in **(D–F)**. Two-sample *t*-test shows that the difference is significant (*t*-Statistic = 1.981, *p* > *t* = 0.03). **(D–F)** No change in preference for multiple flavor pairs without STFP. Bas, Basil; Min, Mint; Co, Cocoa; Cin, Cinnamon; Cu, Cumin; Man, Dry Mango; T1—test 1 representing animals with preference <0.5, T2—test2 representing change in preference from T1 for corresponding animals; T1-T2 intervals: 1 day for basil vs. mint, 4 days for cumin vs. dry mango, 14 days for cocoa vs. cinnamon. Gray circles and connecting dashed lines represent preference trend for an individual mouse. Error bars represent standard error of the mean; n.s.—no significance. Gray bars represent the means with error bars representing SEM, gray dots represent individual animals. ^*^represents *p* ≤ 0.05, ^**^represents *p* ≤ 0.01.

In order to establish our modified design as viable method for studying STFP, we first used cocoa and cinnamon flavor pair. Animals were presented with the flavored food in two cups simultaneously in the experimental cage. Preference for each flavor was estimated as a ratio of W_*C*.*F*_ for respective flavor vs. total food consumed. The pre-STFP preference was estimated 24 h before the demonstration. Based on the pre-STFP preferences, next day we fed a DemoMice on less preferred flavor and released him in the home cage of corresponding ObMice for an hour long session of social interaction. Post-STFP preference tests were conducted 24 h and 16 days after the social interaction (demonstration). These tests were conducted as explained in the methods section. For 24 h post-STFP preference test conducted with 15 mice, 8 mice show an increase in demoFlavor preference post-STFP with least change being 0.02 and the largest change being 0.83. 4 out of 15 mice show decrease in preference while 3 out of 15 mice did not show any change from zero preference for demoFlavor within experimental errors. Using W_C.F_ as the read out (Figure 2A), we find that observers consumed more of demoFlavor after interaction. Means were calculated using two approaches (i) by including all the animals (*n* = 15) and, (ii) by excluding the animals with no consumption during both the testing sessions (*n* = 12, not shown in plots). For *n* = 15, 24 h post-STFP preference for demoFlavor (0.41 ± 0.10) was found to be higher than that for pre-STFP test (0.16 ± 0.06). For *n* = 12, we observe increased difference between pre-STFP vs. 24 h post-STFP preferences (pre-STFP preference = 0.20 ± 0.07, 24 h post-STFP preference = 0.51 ± 0.11). The numbers are presented as Mean ± SEM. Significance was calculated using paired-sample *t*-test to evaluate the effect of social demonstration on change in preference (by design, the flavor with low preference during pre-STFP test or first test is chosen to be the demonstrated flavor). Increase in preference seen during post-STFP test was found to be significant irrespective of considering or not considering the animals with zero consumption. Paired sample *t*-test: *n* = 15, *t*-Statistic = −3.11, *p* > |t| = 0.008 (when considering all); *n* = 12, *t*-Statistic = −3.39, *p* > |t| = 0.006 (excluding the animals that showed zero consumption). We show that social interaction significantly increases the preference for demoFlavor.

#### Change in preference without demonstration

Though our design allows for enhancing the dynamic range for measuring the preferences, it is important to establish that the pre-STFP preferences are reflective of innate preference and they are stable across repeated measurements. In order to test this, we compared relative preferences for multiple flavor pairs without intervening demonstration across two tests separated by multiple days (Figures 2D–F). We found that without demonstration, preference for the tested flavors did not change across 1 day (basil 0.7 vs. mint 1% flavored food), 4 days (cumin 0.5% vs. dry mango 1% flavored food) or 14 days (cocoa 2% vs. cinnamon 1% flavored food, same flavor pair that is used in experiment) suggesting that the pre-STFP preferences had a dominant contribution from innate preference. In our modified STFP design, we note that pre-assignment of low preferring mice could possibly result in a biased scenario of detecting a positive change if the preferences were purely random. This is so because the probability of post-demonstration increase in preference becomes dependent on the starting preference (i.e., pre-STFP preference) if the preferences were displayed due to random chance. We tested this in two ways:

- Through Analysis of Experimental Observation:
  We plotted the observed change in preference as a function of starting preference (Figure 2B). For both the groups i.e., one tested with demonstration and another tested without demonstration, we found that the correlation between the initial preference vs. change in preference was very low (Figure 2B; Mice tested with STFP—(black squares) Intercept = 0.34 ± 0.09, Slope = −0.23 ± 0.35; Mice tested without STFP—(gray circles) Intercept = 0.22 ± 0.12, Slope = −0.30 ± 0.38). Further, we selected the aforementioned three groups of non-demonstrated animals based on test 1 preference of <0.5 [Figures 2D–F: Bars T1 (*Pr* < 0.5) and T2 for all three flavor pairs]. We then followed same animals and looked at the change in preference during test 2 without intervening demonstration. Even after sorting animals in similar manner as done in our modified STFP design, we find that preference did not change without demonstration [Mean Preferences: Basil vs. Mint T1 = 0.27 ± 0.04, T2 = 0.44 ± 0.09, *F*_(1, 18)_ = 2.76, *p* > *F* = 0.11; Cumin vs. Dry Mango T1 = 0.29 ± 0.05, T2 = 0.48 ± 0.08, *F*_(1, 18)_ = 4.08, *p* > *F* = 0.06; Cocoa vs. Cinnamon T1 = 0.30 ± 0.04, T2 = 0.32 ± 0.10, *F*_(1, 18)_ = 0.031, *p* > *F* = 0.86]. Finally, when we compare change in preference for demonstrated group (*n* = 23: 12 mice from cocoa-cinnamon group and 11 mice from basil-thyme group) and non-demonstrated group (*n* = 29: 10 animals each from the three non-demo groups excluding one animal with zero consumption during both tests) we find that the increase in preference for demonstrated group is higher than that of non-demonstrated group across two tests (Figure 2C; Mean change in preference: Demo Group = 0.294 ± 0.067, No Demo Group = 0.131 ± 0.049). Two sample *t*-test shows that the difference in the means is significant (*t*-Statistic = 1.981, *p* > *t* = 0.03). In essence, our data for modified STFP paradigm suggests that this protocol can be used to study STFP and is consistent with previous studies done using similar protocols.
- Comparison through simulation—Random vs. Selective demonstration
  For understanding the effect of innate preference in our new method of selecting the “demoFlavor” based on pre-STFP preferences, we simulated the measurement of change in preference as follows: We simulated a scenario of two successive preference tests, corresponding to pre-STFP and post-STFP tests being conducted with *N* number of animals with a flavor pair A and B. The preferences for individual animals are distributed around the mean 0.5 (equal preference for both the flavors). To achieve this, we generated the following three sets of random numbers with a mean 0.5 and a fixed standard deviation (used as test parameter in our case): (i) Set SP—Each value in this set represents the starting preference (SP) component to estimate demoFlavor preference for one animal, say, animal X, (ii) Set RP1—Corresponding value represents random preference component to estimate demoFlavor preference of animal X during test 1, and (iii) Set RP2—Corresponding value represents random preference component that is independent of innate preference to estimate demoFlavor preference of animal X during test 2. We arrived at the preferences exhibited by each of the animals in two of the tests corresponding to pre-STFP and post-STFP as follows: The preferences Pi, in general, are considered as a sum of contribution from initial preference component and random choice component weighted by a parameter “i.” This parameter represents the magnitude of influence of the innate preference for a given animal.

We simulated the two scenarios for observing the effect of innate preference on change in preference, first scenario (random demonstration) being the case when half of the animals are demonstrated with either of the flavors after first test, while the second scenario (selective demonstration) being the case when animals with <0.5 preference are chosen to be demonstrated with less preferred flavor. From these three sets, we calculated P1 and P2 i.e., preference for demoFlavor after test 1 and 2, respectively, as follows:

$$\begin{array}{lll} P1\; & = & \;i\;\cdot \;IP\;+\;\left(1\;-\;i\right)\;\cdot \;RP1,\\ P2\; & = & \;i\;\cdot \;IP\;+\;\left(1\;-\;i\right)\;\cdot \;RP2\;and\\ \Delta P\; & = & \;P2\;-\;P1 \end{array}$$

where, *i* represents the weight of *IP* on *P1* or *P2* and Δ*P* represents change in preference across test 1 and test 2.

From this dataset, in order to simulate random demonstration scenario, we sorted P1, P2 pairs in ascending order of P1 following which we selected every alternate animal to estimate the mean Δ*P*. Alternatively, to simulate the selective demonstration scenario, we used P1-P2 pairs and arranged for P1 values in the range 0 < P1 < 0.5 to arrive at Δ*P* w.r.t *IP*. The simulation showed that the difference in Δ*P* seen in both the simulated scenarios is a function of the standard deviation of the distributions and more importantly the parameter “*I*” (data not shown). However, importantly with a dominant innate preference (0.5 < *i* < 0.95), the difference between both the groups are negligible. We find that the average change in preference, < Δ*P* > for both the random and selective demonstration scenarios was similar across the chosen range of innate preference (For *i* = 0.6: Δ*P*_*Random demo*_ = −0.00055 ± 0.00517, Δ*P*_*Selective demo*_ = 0.0165 ± 0.00514; For *i* = 0.95: Δ*P*_*Random demo*_ = −0.00078 ± 0.00064, Δ*P*_*Selective demo*_ = −0.00014 ± 0.00064; assuming 10% standard deviation).

Having established the modified STFP-paradigm to show recent retrieval of STFP memory using cocoa and cinnamon flavors, next we wanted to study the ability of the animal to discriminate similar flavors during short and long term testing. One aspect of remote retrieval is memory generalization and it can be tested using STFP with perceptually similar flavor pairs. Multisensory flavor perception is considered largely to arise from a combination of gustatory and olfactory perception of food (Spence, 2015). On this basis, flavoring agents with odor imparting organic molecules from chemically diverse families (e.g., cocoa and cinnamon) can be considered relatively more different in their flavor perception in comparison to those flavoring agents which contain chemically similar odor imparting organic molecules (e.g., thyme and basil). We wanted to establish if animals can discriminate and retain their preference for the demoFlavor even after consolidation when the demoFlavor and non-demoFlavor are perceptually similar. We choose thyme and basil flavored food as similar flavors since they are perceptually similar based on their chemical composition. We tested the animals for their preferences 24 h, 17-, and 41 days following training in an effort to observe recent as well as remote retention of STFP based discrimination between qualitatively similar flavors.

#### Thyme vs. basil flavored food

**Figure 3:**
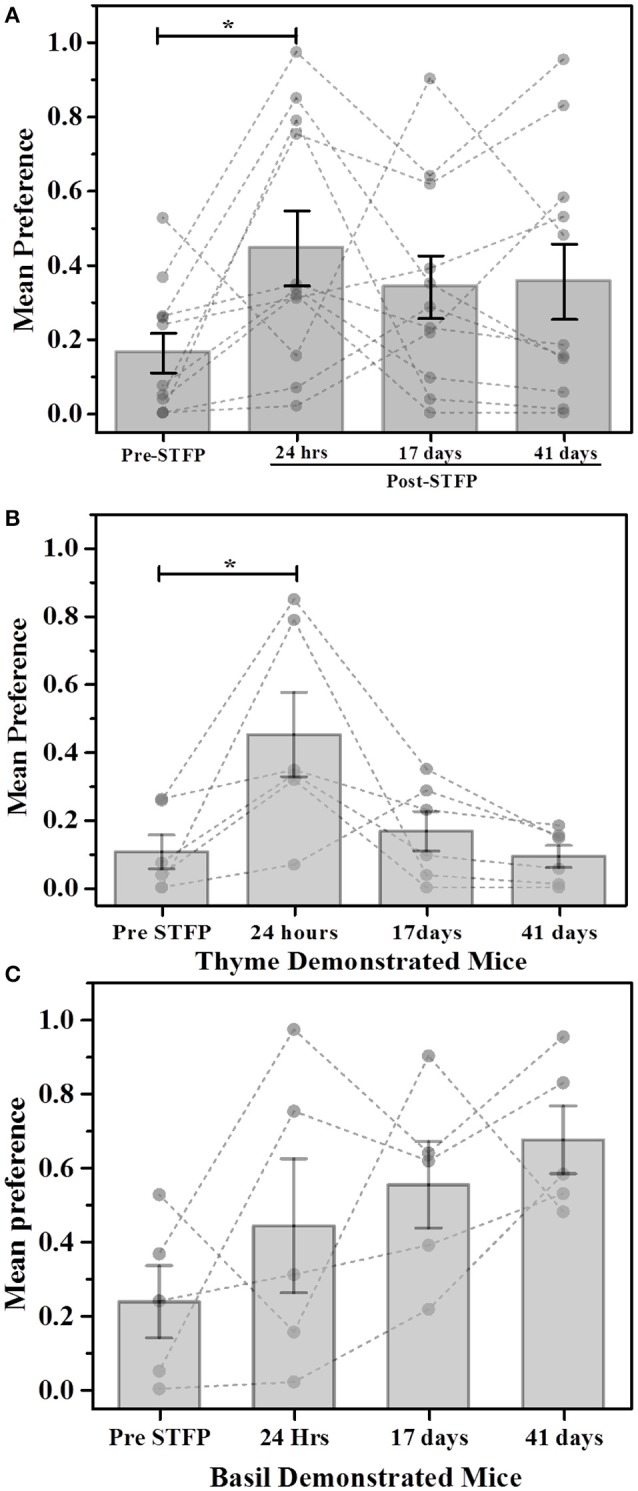
**Weight based evaluation of preferences for thyme and basil demonstrated mice. (A)** Mice were presented with thyme and basil flavored food for preference tests conducted before and after STFP (24 h, 17- and 41 days post-STFP). Each bar represents mean preference for demonstrated flavor (*n* = 11). Gray dots and connecting dotted lines represent preference trend for an individual mouse. Significance was calculated using 1W-RM-ANOVA at 0.05 level of significance. 24 h post-STFP preference was relatively higher (preference = 0.45 ± 0.10) in comparison to pre-STFP [preference = 0.16 ± 0.05; Wilks' lambda = 0.35; *F*_(3, 8)_ = 5.004, *p* > *F* = 0.03; Tukey's test: pre-STFP vs. 24 h: *t*-statistic = 4.01, *p* = 0.038]. 17 days (preference = 0.34 ± 0.08) and 41 day post-STFP preference (preference = 0.36 ± 0.10) is not significantly higher as compared to pre-STFP. **(B)** Thyme DemoMice (*n* = 6): Significant increase in demoFlavor preference is observed only for 24 h post-STFP test while 17 day and 41 day preferences are similar to pre-STFP values. Difference between 24 h, 17 day, and 24 h, 41 day preference was also significant. **(C)** Basil demo mice (*n* = 5): Increasing trend of preferences across successive testing sessions is observed. Mean comparison that population means are not significantly different. Significance was calculated using 1W-RM-ANOVA with *post-hoc* analyses. “^*^” indicates *p* < 0.05; Vertical bars (Gray) indicate mean preferences for corresponding testing sessions; error bars indicate standard error of mean.

Using W_C.F_ as the read out for STFP conducted with thyme—and basil flavored food (Figure 3), we observed an average pre-STFP preference (*n* = 11) of 0.16 ± 0.05 for less-preferred flavor. After STFP, the preference of flavors is measured and we observed an increase in preference of the demoFlavor to 0.45 ± 0.10) from the pre-STFP levels. This trend was observed for preference tests conducted after 17 days (preference = 0.34 ± 0.08) as well as after ~6 weeks (41 days) of STFP (preference = 0.36 ± 0.10). 1W-RM-ANOVA was conducted to evaluate the difference between pre- and post-STFP mean preference for demoFlavor. ANOVA indicated a significant difference at *p* < 0.05 level among these preferences [Wilks' lambda = 0.35, *F*_(3, 8)_ = 5.004; *p* > *F* = 0.03]. Further comparison of means using *post-hoc* analysis revealed that 24 h preference is significantly higher than pre-STFP whereas 17- and 41 day preference is not significantly higher as compared to pre-STFP (Tukey Test; pre-STFP vs. 24 h: t-statistic = 4.01, *p* = 0.038). 24 h preference was also significantly different from 17- and 41 day test preferences (24 h vs. 17 day test: t-statistic = 4.21, *p* = 0.004; 24 h vs. 41 day test: *t*-statistic = 5.31, *p* = 0.009). This result suggests that animals do not retain flavor-safety associations formed during STFP for long term and remote discrimination between similar flavors is lost with time. During this experiment, five out of 11 mice were demonstrated with basil flavored food while remaining six mice were demonstrated with thyme flavored food. In order to analyze flavor specific retention of preferences, we analyzed the weight data for these two sub-groups individually (Figures 3B,C).

For thyme DemoMice (Figure 3B), we observed an increase in preference after 24 h of STFP (*n* = 6; Pre-STFP preference = 0.10 ± 0.05, 24 h preference = 0.45 ± 0.12). This preference change was not retained for 17- and 41 day preference tests for thyme DemoMice. 1W-RM-ANOVA was conducted to evaluate the effect of social interaction on preference change. Mauchy's test indicated that the assumption of sphericity was violated (χ^2^ = 11.52, *p* = 0.04). This violation was compensated by performing Greenhouse-Geisser correction (ε = 0.41) to degrees of freedom. The final analysis indicated that the population means were significantly different at 0.05 significance level [*F*_(3, 1.223)_ = 6.18, *p* > *F* = 0.04]. Further comparison of means using *post-hoc* analyses revealed that pre-STFP preference was significantly different only from 24 h data while 17- and 41 day preference was similar to the pre-STFP levels (Tukey's *post-hoc* mean comparison at 0.05 significance level; pre-STFP vs. 24 h post-STFP: *p* = 0.01, *t*-Statistic (15) = 5.11, significant;. pre-STFP vs. 17 days post-STFP: *p* = 0.92, t-Statistic = 0.91, non-significant; 41 days vs. pre-STFP: *p* = 0.998, *t*-Statistic = 0.20, non-significant).

For basil DemoMice (Figure 3C), preference for demoFlavor was observed to follow an increasing trend (*n* = 5; Pre-STFP preference = 0.24 ± 0.097, 24 h preference = 0.44 ± 0.18, 17 day preference = 0.55 ± 0.12, and 41 day preference = 0.67 ± 0.09). 1W-RM-ANOVA was conducted to evaluate the effect of demonstration on change in preference. Mauchy's test indicated that the assumption of sphericity was valid and no correction was required for degrees of freedom (χ^2^ = 4.81, *p* > χ^2^ = 0.44). Final analysis revealed that population means were not significantly different at 0.05 level of significance [Wilks' lambda = 0.08, *F*_(3, 2)_ = 3.21; *p* > *F* = 0.06]. In the above analysis, lack of significance could be either a result of mice not retaining the STFP memory or could simply be due to lack of sensitivity of the weight measure.

### Preference estimation by time

#### Total time

**Figure 4:**
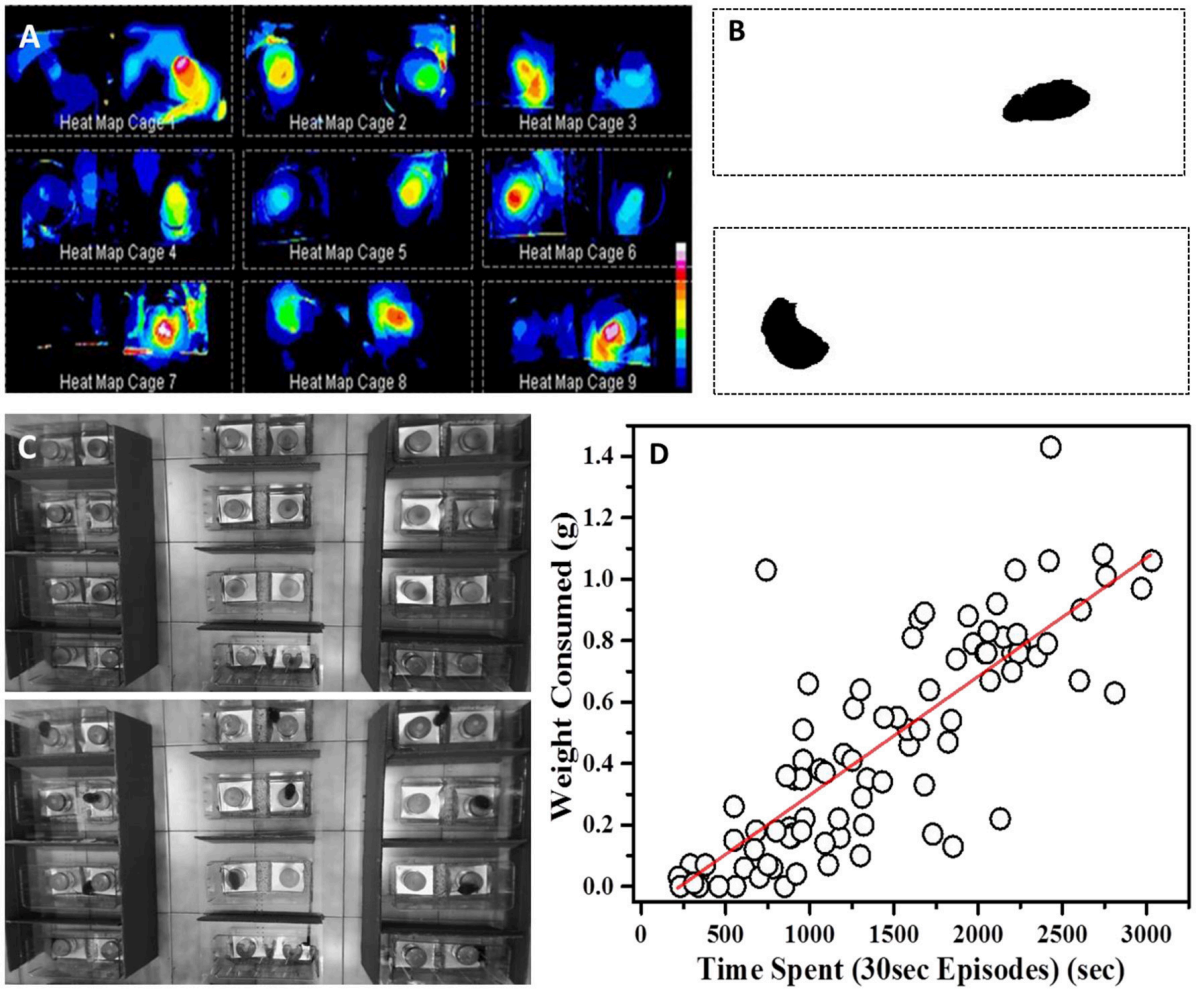
**Time based evaluation of preference (A)** Heat maps of nine representative cages showing temporal preference zones around the food container in 24 h post-STFP preference test. Inset: Scale representing decreasing intensities (vertically downwards) using different colors, white being the highest intensity and blue being the lowest. **(B)** Processed frames after background subtraction and threshold setting. The dark pixels represent position of mouse within the cage. **(C)** Top—Representative background frame Bottom—Representative frame from which background was subtracted to generated heat maps. **(D)** Correlation between amount of food eaten from a cup (y-axis—W_C.F_ in grams) and time spent by a mouse near the same food cup (x-axis—total time spent in seconds). While calculating total time spent, only those seconds are accounted for when an animal continuously spent 30 s or longer at a location. Time and weight information from both cups in each experimental cage was considered for this plot. Each circle represents one food cup. Pearson's *r* = 0.83.

As discussed earlier, measuring W_*C*.*F*_ is very error prone and may not be sensitive enough to pick up small changes. As an alternative, we decided to video monitor and analyze the retrieval tests for the changes in residence time and consumption pattern. Using ImageJ software to process the video, we first generated heat maps for experimental cages which represent differential amounts of time spent near food containers as a function of pixel-intensity (Figures 4A–C). The heat maps were suggestive of the fact that total time spent could be a measure of food consumed in this task.

**Figure 5:**
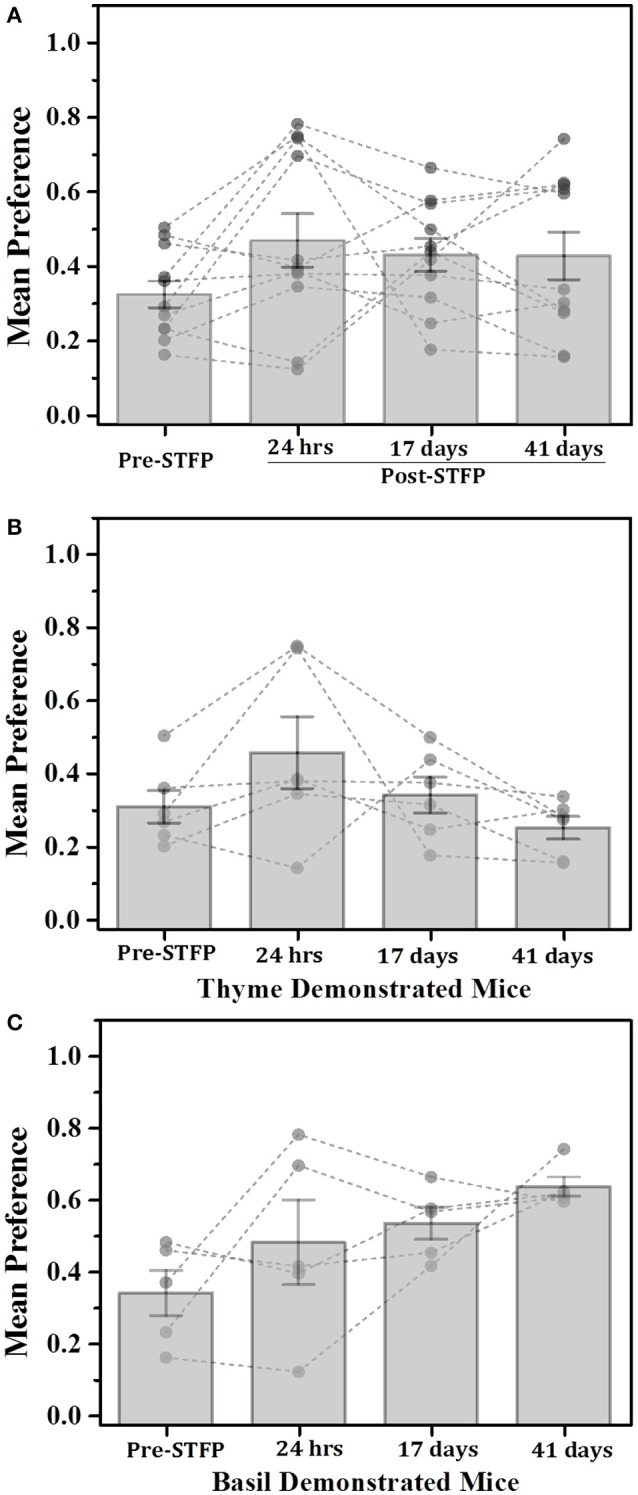
**Total time based comparison of preferences for thyme and basil demonstrated mice. (A)** Plot of mice preference pre- and post-STFP based on manual scoring of time spent near food container with demonstrated flavor. Mice were presented with thyme and basil for preference tests conducted at different time points over a period of 41 days. Pre-STFP preference = 0.32 ± 0.04; 24 h post-STFP preference = 0.47 ± 0.07; 17 days post-STFP preference = 0.43 ± 0.04; 41 days post-STFP preference = 0.42 ± 0.06. Significance was calculated using 1W-RM-ANOVA at 0.05 level of significance. No population means were significantly different from pre-STFP preference [Wilks' lambda = 0.51, *F*_(3, 8)_ = 2.56, *p* > *F* = 0.13]. **(B)** Thyme DemoMice (*n* = 6): Segregated data for only thyme DemoMice. Preference trend indicated decrease during remote retrieval at 17- and 41 day tests. Pre-STFP preference = 0.31 ± 0.04; 24 h post-STFP preference = 0.46 ± 0.10; 17 days post-STFP preference = 0.34 ± 0.05; 41 days post-STFP preference = 0.25 ± 0.03. No population means were significantly different from pre-STFP preference: Wilks' lambda = 0.19, *F*_(3, 3)_ = 4.01, *p* > *F* = 0.14. **(C)** Basil DemoMice (*n* = 5): Increasing trend of preferences across successive testing sessions is observed. Pre-STFP preference = 0.34 ± 0.06; 24 h post-STFP preference = 0.48 ± 0.12; 17 days post-STFP preference = 0.53 ± 0.04; 41 days post-STFP preference = 0.64 ± 0.03. Mean comparison that population means are not significantly different [Wilks' lambda = 0.22, *F*_(3, 2)_ = 2.34, *p* > *F* = 0.31]. Significance was calculated using 1W-RM-ANOVA with *post-hoc* analyses where required. “^*^” indicates *p* < 0.05; Vertical bars (Gray) indicate mean preferences for corresponding testing sessions; error bars indicate standard error of mean. Gray dots and connecting lines represent preference trend for an individual mouse.

We wanted to test if total time spent on top of a food container could be used as a measure of food consumption. We compared the total time spent on top of the demoFlavor container across successive testing sessions (Figure 5A). We then followed it up with a demoFlavor-based segregation of experimental mice and analyzing their preference changes (Figures 5B,C). In doing so, we find that one shot measurement of total time is not any better than the weight measure as described below.

For thyme DemoMice (Figure 5B), we observed an increase in preference after 24 h of STFP (*n* = 6; Pre-STFP preference = 0.31 ± 0.04; 24 h post-STFP preference = 0.46 ± 0.10). This preference change was not retained for 17- and 41 day preference tests for thyme DemoMice. 1W-RM-ANOVA was conducted to evaluate the effect of social interaction on preference change. The final analysis indicated that the population means were not significantly different at 0.05 level of significance [Wilks' lambda = 0.19, *F*_(3, 1.24)_ = 2.39, *p* = 0.11]. For basil DemoMice, preference for demoFlavor was observed to follow an increasing trend (Figure 5C) with smaller error bars in comparison to weight based comparison (Figure 3C). 1W-RM-ANOVA revealed that population means were not significantly different at 0.05 level of significance [Wilks' lambda = 0.22, *F*_(3, 2)_ = 2.34, *p* > *F* = 0.31].

During these estimates, we noticed that in many instances mice spent time on top of the food container without actually engaging in food consumption. Observation of multiple videos revealed that mice spend relatively longer stretches of time (~few tens of seconds) when they are actually eating, henceforth referred to as “episodes.” Mice were also found to be eating either directly from the container or from the food spilled around the container on the aluminum tray. Considering these aspects, we moved on to estimate the preference using cumulative episodes.

#### Preference estimation by eating episodes

As mentioned earlier, while scoring the test videos, we considered continuous stretches of ~30 s or longer near one cup as eating episodes. Using this information, we constructed consumption profile for each mouse. For our analyses, we used cumulative number of such episodes as an indirect measure of food consumption. As a first test, we checked if these episodes are correlated with consumption of food measured through weight. We manually scored preference test videos and plotted the number of episodes for different animals with that of the amount of food consumed (Figure 4D). We observed a strong correlation (Pearson's *r* = 0.83) between the weight of food consumed from a container and the number of episodes near the same container suggesting that one can use it as a proxy for food consumption.

#### Temporal dynamics of food consumption

Based on the strong correlation between W_C.F_ and time spent near the food containers, we used the food consumption episodes to construct cumulative food consumption profiles. This measure provides an added advantage of continuously visualizing temporal dynamics of food consumption by the animal in each session. We note that such a measure is not possible through any single point measurements such as total weight or total time spent. The resulting cumulative consumption profiles corresponding to different tests were modeled using the integral of Weibull function (hereafter referred to as Weibull Cumulative Distribution Function or Weibull CDF). Fitting the time profiles to Weibull CDF as explained, we next compared the dynamics of animal performance for tests conducted before and after 24 h, 17- and 41 days of STFP.

#### Cumulative time profile (CTP) for demonstrated flavor

##### Thyme demonstrated mice

**Figure 6:**
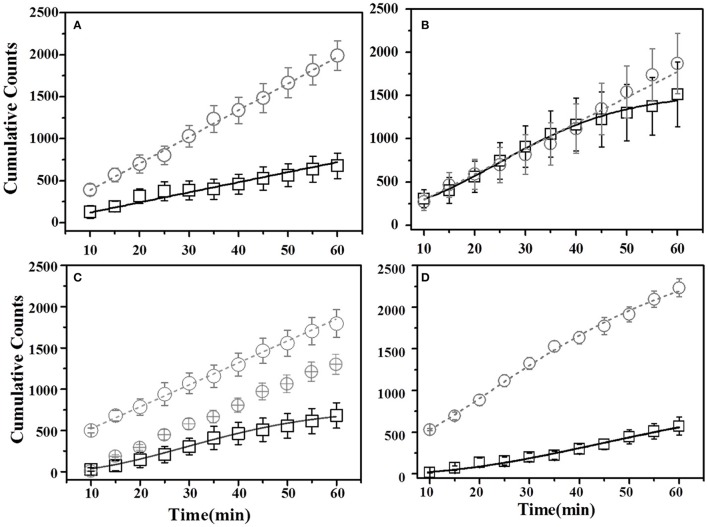
**Cumulative Time profiles (CTPs) for Thyme demonstrated mice**. Open squares represent cumulative consumption of demonstrated flavor (Thyme), open circles represent non-demonstrated flavor (Basil). Black solid curves consistently represent the fit for demoFlavor, gray dashed curve for non-demoFlavor. X-axis represents the total time in minutes. Y-axis represents number of seconds spent as eating episodes near respective food cups. **(A)** Pre-STFP preference test to determine group difference in the relative preference for the flavor pair. Fitting curves for both demo- and non-demoFlavor CTPs represent best approximated fit to a straight line. **(B)** 24 h post-STFP preference test shows change in demoFlavor preference (Thyme; Open squares) in comparison to non-demoFlavor (Basil; Open circles). Fitting curves indicate demoFlavor CTP modeled by Weibull CDF fit while non-demoFlavor CTP is the best approximation to a straight line. **(C)** 17 days post-STFP preference test showing baseline-shifted consumption profile for non-demoFlavor (Basil; Faint-gray encircled crosshairs) in order to highlight the difference between consumption rates for demonstrated and non-demoFlavor. Fitting curves indicate demoFlavor CTP modeled by Weibull CDF fit while non-demoFlavor CTP is the best approximation to a straight line. **(D)** 41 days post-STFP preference test data showing consumption profiles similar to pre-STFP for thyme DemoMice. Fitting curves indicate non-demoFlavor CTP modeled by Weibull CDF fit while demoFlavor CTP is the best approximation to a straight line. Black solid line—represents the fit for demoFlavor CTP, Gray dashed line—fit for non-demoFlavor CTP).

For thyme DemoMice, cumulative time spent near thyme-flavored food was consistently lower as compared to that for basil during 1 h long pre-STFP test session (Figure 6A). CTPs for both thyme and basil were best approximated by a straight line for pre-STFP test. As steeper change in slope represents high rate of consumption in comparison to gradual slope, these time profiles show that these animals consumed more of basil flavored food than thyme. Consistently higher preference for basil during 1 h long pre-STFP testing session is reflected in the time profile. In combination with the initial and final cumulative counts, the rates of consumption for demoFlavor and non-demoFlavor were found to be different [pre-STFP thyme CTP: Linear fit slope = 10.71 ± 0.68, intercept = 46.65 ± 20.75, amplitude = 642.6 ± 40.8; pre-STFP basil CTP: Linear fit slope = 31.75 ± 0.56, intercept = 71.02 ± 16.24, amplitude = 1905 ± 33.6; *F*-Test: *F*_(2, 18)_ = 1111.9, *p* > *F* ≈ 0]. Table 1 summarizes the various fit parameters obtained for tests conducted at 24 h, 17- and 41 days after demonstration.

**Table 1:** **Summary of curve fitting for Thyme demonstrated mice**.

					**N**	**Weibull CDF/linear–fit parameters**				**Fit statistics**	
						**A**	**B**	**d/intercept**	**k/slope**	**Reduced χ^2^**	**Adjusted *R*^2^**
Thyme Demonstrated Mice	Demonstrated	Flavor	Pre-STFP	Linear Fit	6	642.6 ± 40.8	–	46.65 ± 20.75	10.71 ± 0.68	0.982	0.961
			24 h Post-STFP	Weibull Fit	6	1322.02 ± 11.76	190.04 ± 40.45	1.96 ± 0.24	0.028 ± 0.0016	0.016	0.996
			17 days Post-STFP	Weibull Fit	6	686.54 ± 32.2	0 ± 0	2.32 ± 0.10	0.026 ± 0.001	0.81	0.997
			41 days Post-STFP	Linear Fit	6	588 ± 22.2	–	−82.28 ± 5.95	9.8 ± 0.37	0.993	0.986
	Non-Demonstrated	Flavor	Pre-STFP	Linear Fit	5	1905 ± 33.6	–	71.02 ± 16.24	31.75 ± 0.56	0.993	0.997
			24 h Post-STFP	Linear Fit	5	1840.8 ± 60	–	−35.56 ± 27.43	30.68 ± 1.006	0.995	0.989
			17 days Post-STFP	Linear Fit	5	1597.2 ± 107.4	–	243.51 ± 39.16	26.62 ± 1.79	0.998	0.996
			41 days Post-STFP	Weibull Fit	5	2411.03 ± 401.56	296.4 ± 62.16	1.51 ± 0.21	0.02 ± 0.003	0.20	0.998

Further, comparison of amplitude parameter for all the CTP fits also revealed that STFP mediated change in preference is retained only until recent test for thyme DemoMice (**Figure 8A**). Comparison of amplitudes using one way ANOVA shows significance between the means [*F*_(3, 29)_ = 34.42, *p* < 0.0001]. *Post-hoc* analysis reveals that pre-STFP amplitude for cumulative consumption was significantly different only from 24 h mean at 0.05 level of significance (pre-STFP amplitude = 642.6 ± 40.8, 24 h amplitude = 1322.02 ± 111.76, *p* < 0.0001) and not from 17- and 41 day amplitude means (17-day amplitude = 686.5 ± 32.2, *p* = 0.94; 41 day amplitude = 588 ± 22.2, *p* = 0.88).

##### Basil demonstrated mice

**Figure 7:**
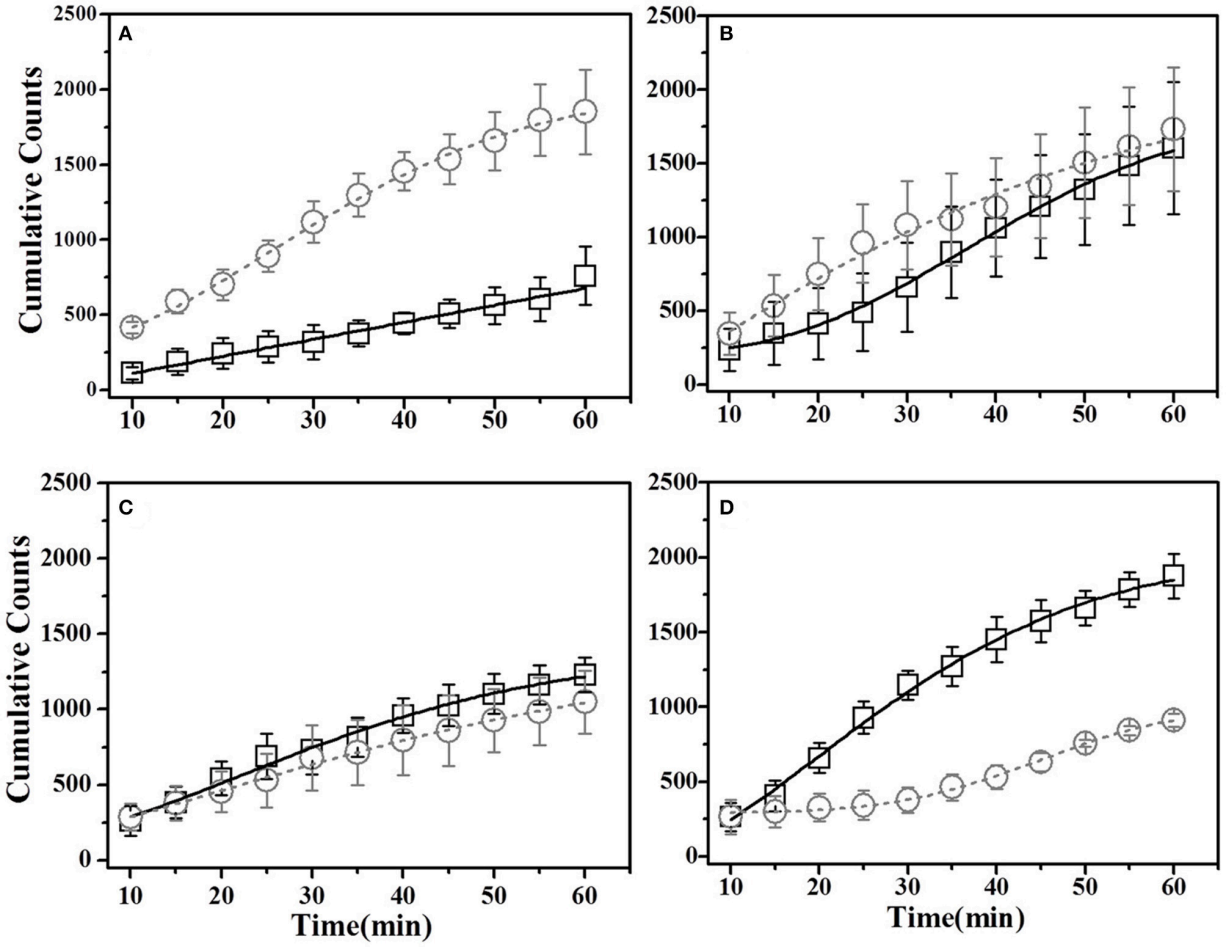
**Cumulative Time profiles (CTPs) for Basil demonstrated mice**. Open squares represent cumulative consumption of demonstrated flavor (Basil), open circles represent non-demonstrated flavor (Thyme). **(A)** Pre-STFP preference test to determine group difference in the relative preference for the flavor pair. Fitting curves indicate non-demoFlavor CTP modeled by Weibull CDF fit while demoFlavor CTP is the best approximation to a straight line. **(B)** 24 h post-STFP preference test shows change in demoFlavor preference (Basil; Black open squares) in comparison to non-demoFlavor (Thyme; open circles). Fitting curves indicate Weibull CDF fits for both the demoFlavor and non-demoFlavor CTP. **(C)** 17 days post-STFP preference test showing retention of STFP memory in terms of dynamics of food consumption which different from pre-STFP test for these Basil DemoMice. Fitting curves indicate Weibull CDF fits for both the demoFlavor and non-demoFlavor CTP. **(D)** 41 days post-STFP preference test data showing consumption profiles almost opposite to that of pre-STFP for Basil DemoMice. This represents retention of remote STFP-memory for Basil DemoMice. Fitting curves indicate Weibull CDF fits for both the demoFlavor and non-demoFlavor CTP. Black solid line—represents the fit for demoFlavor CTP, Gray dashed line—fit for non-demoFlavor CTP.

For this sub-group of mice, cumulative time spent near basil-flavored food is lower as compared to that for thyme during pre-STFP test (Figure 7A). Cumulative counts were consistently higher for thyme flavored food during 1 h long session in comparison to basil. These time profiles represent that thyme flavored food was preferred over basil (pre-STFP—Basil CTP: Linear fit slope = 11.21 ± 1.66, intercept = 3.9 ± 49, amplitude = 672.6 ± 99.6; Thyme CTP: Weibull CDF fit amplitude = 1715.18 ± 173.5, *b* = 283.56 ± 38.08, *d* = 1.88 ± 0.21, *k* = 0.026 ± 0.002). Table 2 summarizes the various fit parameters obtained for tests conducted at 24 h, 17-, and 41 days after demonstration.

**Table 2:** **Summary of curve fitting for Basil demonstrated mice**.

					**N**	**Weibull CDF/Linear–Fit Parameters**				**Fit Statistics**	
						**A**	**B**	**d/intercept**	**k/slope**	**Reduced χ^2^**	**Adjusted *R*^2^**
Basil Demonstrated Mice	Demonstrated	Flavor	Pre-STFP	Linear Fit	5	672.6 ± 99.6	–	3.9 ± 49	11.21 ± 1.66	0.996	0.991
			24 h Post-STFP	Weibull Fit	5	1583.5 ± 199.97	211.42 ± 26.68	2.5 ± 0.30	0.022 ± 0.0019	0.015	0.994
			17 days Post-STFP	Weibull Fit	5	1660.48 ± 184.6	0 ± 0	1.14 ± 0.07	0.021 ± 0.0037	0.03	0.996
			41 days Post-STFP	Weibull Fit	5	2026.87 ± 75.82	0 ± 0	1.64 ± 0.08	0.028 ± 0.0018	998.36	0.997
	Non-Demonstrated	Flavor	Pre-STFP	Weibull Fit	6	1715.18 ± 173.5	283.56 ± 38.08	1.88 ± 0.21	0.026 ± 0.002	0.058	0.997
			24 h Post-STFP	Weibull Fit	6	1626.39 ± 234.46	211 ± 0	1.63 ± 0.19	0.026 ± 0.004	0.06	0.98
			17 days Post-STFP	Weibull Fit	6	1252.8 ± 368.38	160.95 ± 50.86	1.35 ± 0.28	0.019 ± 0.0059	0.009	0.997
			41 days Post-STFP	Weibull Fit	6	675.5 ± 31.8	294.3 ± 11.66	4.16 ± 0.31	0.02 ± 0.0003	0.03	0.997

*N, Number of animals; A, amplitude parameter estimated from the fit; b, Offset/intercept parameter for Weibull CDF fits, d, deviation parameter representing the deviation of fit from exponential for Weibull CDF indicating the duration for which initial intake rate is maintained, /intercept = represents the intercept for linear fit when Weibull fit parameters ascertained approximation of CTP to a straight line, k = slope of the Weibull fit representing the decline in rate of consumption, /slope = slope of linear fit when Weibull fit parameters ascertained approximation of CTP to a straight line. (Refer Section Curve Fitting)*.

**Figure 8:**
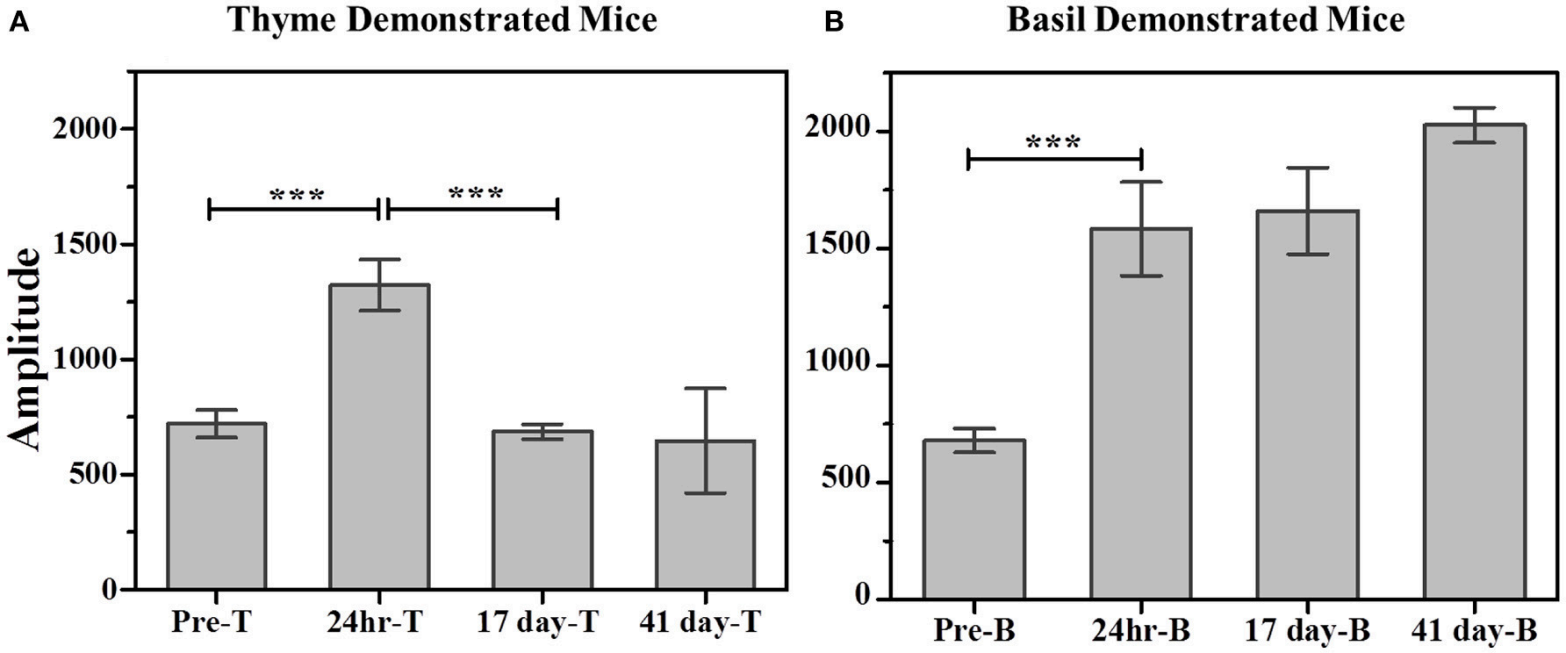
**Comparison of cumulative consumption using amplitude parameter as estimated from fits of the respective cumulative time profiles**. **(A)** Cumulative consumption of thyme for thyme DemoMice. Comparison of amplitudes using one way ANOVA shows significance between the means [*F*_(3, 29)_ = 34.42, *p* < 0.0001]. *Post-hoc* analysis reveals that pre-STFP amplitude for cumulative consumption was significantly different only from 24 h mean (pre-STFP amplitude = 642.6 ± 40.8, 24 h amplitude = 1322.02 ± 111.76, *p* < 0.0001) and not from 17 day and 41 day—amplitude means (17 day amplitude = 686.5 ± 32.2, *p* = 0.94; 41 day amplitude = 588 ± 22.2, *p* = 0.88). **(B)** Cumulative consumption of basil for basil DemoMice. Comparison of amplitudes using one way ANOVA shows significance between the means [*F*_(3, 29)_ = 18.48, *p* < 0.0005]. *Post-hoc* analysis reveals that pre-STFP amplitude for cumulative consumption was significantly different from all other test means (pre-STFP amplitude = 672.6 ± 99.6; 24 h amplitude = 1583.5 ± 199.9, *p* = 0.0005; 17 day amplitude = 1660.48 ± 184.6, *p* = 0.0001; 41 day amplitude = 2026 ± 75, *p* < 0.0001). Bars represent mean amplitude estimated from the fit parameters, error bars represent SEM. Significance calculated at 0.05 level of significance. ^***^indicates *p* < 0.001.

Unlike thyme DemoMice, remote retrieval was evident for STFP mediated change in preference of basil DemoMice (Figures 6D, 7D, 8). The difference in remote cumulative consumption as represented by different CTP parameters for both the flavors showed that animals consume more of demoFlavor than non-demoFlavor [CTP amplitudes for 41 days post-STFP test: demoFlavor = 2026.87 ± 75.82, non-demoFlavor = 675.5 ± 31.8; these amplitudes are significantly different based on ANOVA: *F*_(1, 13)_ = 243.23, *p* > *F* ≈ 0].

Further, comparison of amplitude parameter for all the CTP fits also revealed that STFP mediated change in preference is retained for all the post-STFP tests for basil DemoMice (Figure 8B). Comparison of amplitudes using one way ANOVA shows significant difference between the means [*F*_(3, 29)_ = 18.48, *p* < 0.0005]. Tukey's *post-hoc* analysis revealed that pre-STFP amplitude for cumulative consumption was significantly different from all other test means at 0.05 level of significance (pre-STFP amplitude = 672.6 ± 99.6; 24 h amplitude = 1583.5 ± 199.9, *p* = 0.0005; 17 day amplitude = 1660.48 ± 184.6, *p* = 0.0001; 41 day amplitude = 2026 ± 75, *p* < 0.0001), suggesting that STFP memory for basil DemoMice is retained across 41 days.

Finally, we note that for thyme DemoMice, CTPs for remote tests have equivalent parameters as obtained for its pre-STFP CTP indicating that pattern of consumption is similar in both situations [pre-STFP amplitude = 642.6 ± 40.8, 17 day amplitude = 686.5 ± 32.2, 41 day amplitude = 588 ± 22.2; no significant difference based on ANOVA; *F*_(2, 29)_ = 2.25, *p* > *F* = 0.12]. In this case, absence of remote retrieval for STFP mediated change in preference could be either due to loss of information that was acquired and retrieved at recent time point or it could be due to memory generalization. Based on the CTP fit comparison and finding similar parameters for pre-STFP and 41 day STFP tests, we propose that the flavor-safety association for thyme DemoMice has been lost over time and the observed effect is not due to generalization. Alternatively, difference in pattern of consumption would have indicated generalization of memory in this case. On the other hand, basil DemoMice show significantly different CTPs across successive tests from recent to remote time point indicating retention of acquired associations over time.

## Discussion

Few previous studies have demonstrated retrieval of STFP memories as long as ~4 weeks after training in rodents (Lesburguères et al., 2011). Here, we provide the first evidence of remote STFP memory retrieval in mice after ~7 weeks of training. We show flavor dependent remote retrieval of STFP memory where basil DemoMice retain the STFP mediated change in preference for up to ~7 weeks while thyme DemoMice do not retain the STFP mediated change in preference beyond recent time point (24 h). It is interesting to note that these flavors are similar in their chemical composition (Pavia, 1973; Lee et al., 2005; Satya et al., 2012).

Using weight as a measure for cocoa and cinnamon flavored food in our first experiment, we showed that modified protocol is effective for conducting STFP with mice. Animals consumed significantly higher amounts of food with demoFlavor during 24 h post-STFP preference test in comparison to its pre-STFP consumption (Figure 2A). In parallel, we further addressed the limitation of weight measurement to estimate the change in preference using thyme and basil flavor pair (Figure 3). Differences in consumption profile dynamics are left unobserved while implementing the one-shot measure of total weight and total time to estimate the change in preference. By implementing multiplexed video monitoring set-up as an alternative, we show that cumulative time profiles (CTPs) provide the required sensitivity to measure the STFP induced change in preference along with the additional advantage of continuous monitoring. These improvements in protocol and analyses allowed us to establish CTPs as an alternate measure for monitoring food consumption during the testing sessions. Also, implementation of time as a measure for mean preference can be a way to automate the time profile-based analysis for STFP videos which, in turn, could reduce the amount of time required for STFP using W_C.F_ as a means to calculate mean preference and greatly simplify the procedural tediousness involved in weight measure.

Further comparison of the CTPs obtained from video-based analysis revealed previously unobserved differences between demonstrated and non-demoFlavor consumption before and after STFP. Our results showed that basil DemoMice retain the STFP mediated change in preference for recent test conducted after 24 h of STFP as well as for the remote tests conducted after 17 and 41 days of STFP (Figure 8). This was found not to be holding for remote tests conducted with thyme DemoMice where STFP mediated change in preference resulted in increased consumption of the demoFlavor during recent test conducted after 24 h of STFP but not for remote tests. Regular tests done using the weight as a measurement would not distinguish this aspect of remote retrieval for STFP memory.

### Sensitivity as signal-to-noise ratio (SNR)

Further, we consider the average preference as the measured signal in our experiments while the corresponding standard error of mean is the associated noise with the signal. Sensitivity (Ψ) for our measurements can be defined as a ratio of this signal to corresponding noise. Table 3 summarizes the SNRs for total weight, total time and CTP based estimation of preferences. Average sensitivity (< Ψ >) for each of these measures was calculated as follows:

$$\begin{array}{l} <\text{Ψ}>\;=\;\frac{{\text{Ψ}}_{Pre-STFP}\;+\;{\text{Ψ}}_{24\;h\;}\;+\;{\text{Ψ}}_{17days}\;+\;{\text{Ψ}}_{41days\;Post-STFP}}{4} \end{array}$$

**Table 3:** **Summary of signal to noise ratio for demonstrated flavor as estimated by total weight, total time, and cumulative time profiles**.

		**Total Weight**			**Total Time**			**CTP Amplitude**		
		**S**	**N**	***S/N***	**S**	**N**	***S/N***	**S**	**N**	***S/N***
Thyme DemoMice	Pre-STFP	0.10	0.05	*2.00*	676.90	152.10	*4.45*	642.60	40.8	*15.75*
	24 h post-STFP	0.45	0.12	*3.75*	1516.70	374.80	*4.05*	1322.02	111.76	*11.83*
	17 days post-STFP	0.16	0.06	*2.67*	681.70	154.70	*4.41*	686.50	32.2	*21.32*
	41 days post-STFP	0.09	0.03	*3.00*	573.30	107.50	*5.33*	588.00	22.2	*26.49*
Basil DemoMice	Pre-STFP	0.24	0.10	*2.47*	762.00	193.20	*3.94*	672.60	99.60	*6.75*
	24 h post-STFP	0.44	0.18	*2.44*	1604.00	447.20	*3.59*	1583.50	199.90	*7.92*
	17 days post-STFP	0.55	0.12	*4.58*	1230.00	113.40	*10.85*	1660.48	184.6	*9.00*
	41 days post-STFP	0.67	0.09	*7.44*	1876	146.90	*12.77*	2026.00	75.00	*27.01*

*S, signal—average consumption; N, noise—standard error of mean*.

**Figure 9:**
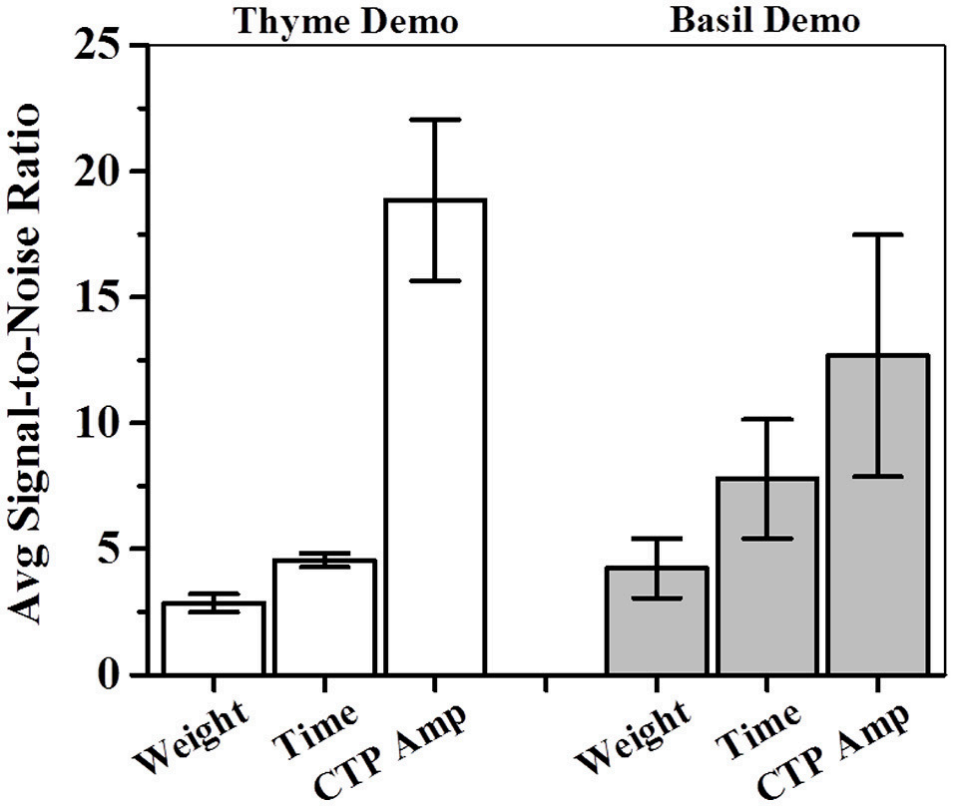
**Average sensitivity or signal-to-noise ratios (SNRs) for total weight, total time and CTP based estimations of consumption amplitudes**. For each measure, average SNR was calculated by adding individual SNR for each of the four successive test sessions (pre-STFP to 41 days) and dividing the sum by 4. CTP based estimations were found to be higher than total weight or total time based estimations of sensitivity. SNR—signal-to-noise ratio calculated for each session by dividing average preference with associated standard error of mean; CTP—cumulative time profile; white bars indicate avg. SNR for thyme DemoMice; white bars indicate avg. SNR for basil DemoMice; error bars indicate S.E.M.

For one-shot measurements of total weight and total time, we observe that average sensitivities (< Ψ_Total Weight_ >: Thyme DemoMice = 2.86, Basil DemoMice = 4.23; and < Ψ_Total Time_ >: Thyme DemoMice = 4.56, Basil DemoMice = 7.79) are lower in comparison to the average sensitivity obtained by comparing amplitudes obtained from fits modeling the CTPs (< Ψ_CTP Amplitude_ >: Thyme DemoMice = 18.85, Basil DemoMice = 12.67), with CTPs consisting of multiple-shot measurements/time bins (Figure 9). For amplitude estimates in Figure 8 (Table 3), we also observe that while the noise is lesser (smaller error bars), the overall trend of change in preference across successive testing sessions is same as observed for single-shot estimates. Thus, lesser noise of the CTP fits allows us to bring out the subtle difference observed between pre- and post-STFP measurements of recent and remote preference as opposed to more noisy one-shot measurements. For basil DemoMice (Figure 8B), we see that the post-STFP preferences for demoFlavor (24 h, 17- and 41 days) are significantly different from the pre-STFP demoFlavor preference while they are not significantly different from each other, in a similar manner as observed with total time or total weight based estimates.

### CTP fits

One of the important developments in our study is the use of CTP fits to arrive at amplitude of food consumption. In previous studies related to meal microstructure analyses (Fox and Byerly 2004), food intake rates have been estimated by fitting the food intake data obtained independently for consecutive time windows to Weibull function of the following exponential form:

$$\begin{array}{l} y=A\cdot e^{-{\left(kx\right)}^{d}} \end{array}$$

In our study, we used cumulative time profiles to observe the intake rates thus the corresponding function would be the Weibull cumulative distribution function (CDF) given by the following integral:

$$\begin{array}{l} \overset{t}{\underset{0}{\int }}A.e^{-{\left(kx\right)}^{d}}\;dt\;=\;A\cdot \left(1-e^{-{\left(kx\right)}^{d}}\right)+b \end{array}$$

The Weibull CDF fits were characterized by four parameters namely: *A—*amplitude parameter representing cumulative consumption, *b*—offset parameter representing the starting preference at the beginning of the session, *d*—deviation parameter representing the deviation of fit from exponential, and *k*—slope parameter representing the decline in rate of consumption. As a consequence of multiple data points used to model CTPs with Weibull CDF, the estimates are more robust representation of the animals' behavior in comparison to single data point obtained for total weight or total time based estimates.

Additionally, for thyme DemoMice, we observe that CTPs for demo- and non-demoFlavor consumption are overlapping for most of the session duration during 24 h test. This indicates more number of switches being made by mice during first 40 min. In comparison, CTPs for pre-STFP, 17-, and 41 day tests show lesser number of switches made between two cups. Enhanced switching behavior post-STFP indicates retention and retrieval of STFP mediated change in preference. This is to note that such differences in the pattern of consumption are not captured by weight based estimation of change in preference. Likewise, for basil DemoMice, co-progression of demo- and non-demoFlavor CTPs during 24 h test indicated high switching behavior unlike pre-STFP test where CTPs are progressing away from each other with more time being spent on the non-demoFlavor cup. Almost parallel co-progression of CTPs for 17 day test indicates retention of preference. For 41 day test, demoFlavor CTP shows higher consumption of the demoFlavor suggesting that CTPs can bring out additional aspects of the pattern of food consumption.

### Modifications in STFP protocol

Even though the overall structure of proposed paradigm is similar to previously reported protocol, there are few key differences. Primarily, no previous protocols implemented pre-STFP preference test for same animals that were longitudinally tested further. Additionally, advantage of the time-profile analyses resides in the fact that time-profiles for pre-STFP preference test could reveal the innate preference among similar flavors even when W_C.F_ for both the flavors is similar. Secondly, the demonstration session has been modified to resemble the STFP as it happens in natural habitat for rodents. DemoMice are not released in a wired enclosure for STFP as reported in the previous studies using rats or mice. We released the DemoMice in respective ObMice home cage without any enclosure which might restrict their free movement followed by stressed behavioral response during demonstration. Further, the habituation and test sessions were conducted in a cage different from home cage of either ObMice or DemoMice avoiding the effect of spatial memory. Having different test cage along with pseudo-randomization allows us to remove spatial-bias component from the animal's performance. Previously, different animal groups were implemented for determining innate preference in order to determine the bias for any of the flavors selected for experiment. This approach functions with an assumption that flavor preferences are homogenous across a population of laboratory bred mice. Our modified version allows us to take into account for the individual variations for flavor-preferences within a population.

### Conclusion

STFP was found to induce long lasting safety associations with chemically diverse as well as similar flavors. Using sensitive time-profile analysis we show that time spent near food containers can provide better estimates of animal performance in remote memory tests as compared to using weight of food consumed. SNR based comparison shows CTP based analysis to be more sensitive in comparison to conventional methods. During remote memory testing, we observe that only basil DemoMice were able to retain the STFP memory. However, remote memory for chemically similar thyme flavor was not retained.

## Author contributions

AS and JB designed the experiments, conducted analyses and wrote the manuscript. AS performed the bulk of experiments and established the experimental setup. SK contributed in performing STFP experiments with Cocoa and Cinnamon flavored food, in establishing experimental setup and in discussions. VS and AD contributed in manual video scoring and discussions.

### Conflict of interest statement

The authors declare that the research was conducted in the absence of any commercial or financial relationships that could be construed as a potential conflict of interest.

## Abbreviations

DemoMice: Demonstrator Mice
ObMice: Observer Mice
W_CF_: Weight of Consumed Food
demoFlavor: Demonstrated Flavor
CTP: Cumulative Time Profile
SC: Systems Consolidation
IP: Innate preference.

## References

[B1] Anagnostaras S. G. Maren S. Fanselow M. S. (1999). Temporally graded retrograde amnesia of contextual fear after hippocampal damage in rats: within-subjects examination. J. Neurosci. 19, 1106–1114. 9920672 10.1523/JNEUROSCI.19-03-01106.1999 PMC6782148

[B2] Barry D. N. Coogan A. N. Commins S. (2016). The time course of systems consolidation of spatial memory from recent to remote retention: a comparison of the immediate early genes Zif268, c-Fos and Arc. Neurobiol. Learn. Mem. 128, 46–55. 10.1016/j.nlm.2015.12.010 26748021

[B3] Choleris E. Clipperton-Allen A. E. Gray D. G. Diaz-Gonzalez S. Welsman R. G. (2011). Differential effects of dopamine receptor D1-type and D2-type antagonists and phase of the estrous cycle on social learning of food preferences, feeding, and social interactions in mice. Neuropsychopharmacology 36, 1689–1702. 10.1038/npp.2011.50 21525863 PMC3138658

[B4] Choleris E. Clipperton-Allen A. E. Phan A. Kavaliers M. (2009). Neuroendocrinology of social information processing in rats and mice. Front. Neuroendocrinol. 30, 442–459. 10.1016/j.yfrne.2009.05.003 19442683

[B5] Countryman R. A. Kaban N. L. Colombo P. J. (2005). Hippocampal c-Fos is necessary for long-term memory of a socially transmitted food preference. Neurobiol. Learn. Mem. 84, 175–183. 10.1016/j.nlm.2005.07.005 16122949

[B6] Fox E. A. Byerly M. S. (2004). A mechanism underlying mature-onset obesity: evidence from the hyperphagic phenotype of brain-derived neurotrophic factor mutants. Am. J. Physiol. Regul. Integr. Comp. Physiol. 286, R994–R1004. 10.1152/ajpregu.00727.2003 15142855

[B7] Frankland P. W. Bontempi B. Talton L. E. Kaczmarek L. Silva A. J. (2004). The involvement of the anterior cingulate cortex in remote contextual fear memory. Science 304, 881–883. 10.1126/science.1094804 15131309

[B8] Frankland P. W. Bontempi B. (2005). The organization of recent and remote memories. Nature Rev. Neurosci. 6, 119–130. 10.1038/nrn1607 15685217

[B9] Galef B. G. Jr. (1977). Social transmission of food preferences : an adaptation for weaning in rats. J. Comp. Physiol. Psychol. 91, 1136–1140. 10.1037/h0077387

[B10] Galef B. G. Mason J. R. Preti G. Bean N. J. (1988). Carbon disulfide: a semiochemical mediating socially-induced diet choice in rats. Physiol. Behav. 42, 119–124. 10.1016/0031-9384(88)90285-5 3368530

[B11] Goshen I. Brodsky M. Prakash R. Wallace J. Gradinaru V. Ramakrishnan C.. (2011). Dynamics of retrieval strategies for remote memories. Cell 147, 678–689. 10.1016/j.cell.2011.09.033 22019004

[B12] Holmes A. Wrenn C. C. Harris A. P. Thayer K. E. Crawley J. N. (2002). Behavioral profiles of inbred strains on novel olfactory, spatial and emotional tests for reference memory in mice. Genes Brain Behav. 1, 55–69. 10.1046/j.1601-1848.2001.00005.x 12886950

[B13] Kim J. J. Fanselow M. S. (1992). Modality-specific retrograde amnesia of fear. Science 256, 675–677. 10.1126/science.1585183 1585183

[B14] Lee S.-J. Umano K. Shibamoto T. Lee G. (2005). Identification of volatile components in basil (*Ocimum Basilicum* L.) and thyme leaves (*Thymus Vulgaris L*.) and their antioxidant properties. Food Chem. 91, 131–137. 10.1016/j.foodchem.2004.05.056

[B15] Lesburguères E. Gobbo O. L. Alaux-Cantin S. Hambucken A. Trifilieff P. Bontempi B. (2011). Early tagging of cortical networks is required for the formation of enduring associative memory. Science 331, 924–928. 10.1126/science.1196164 21330548

[B16] Pavia D. L. (1973). Coffee, tea, or cocoa. a trio of experiments including the isolation of theobromine from cocoa. J. Chem. Educ. 50, 791–792. 10.1021/ed050p791 4747930

[B17] Plucinska K. Strachan L. Peeters D. Platt B. Riedel G. (2012). Social transmission of food preference in C57BL / 6 Mice, in Proceedings of Measuring Behaviour August (Aberdeen), 488–90.

[B18] Restivo L. Vetere G. Bontempi B. Ammassari-Teule M. (2009). The formation of recent and remote memory is associated with time-dependent formation of dendritic spines in the hippocampus and anterior cingulate cortex. J. Neurosci. 29, 8206–8214. 10.1523/jneurosci.0966-09.2009 19553460 PMC6666032

[B19] Ross R. S. Eichenbaum H. (2006). Dynamics of hippocampal and cortical activation during consolidation of a nonspatial memory. J. Neurosci. 26, 4852–4859. 10.1523/jneurosci.0659-06.2006 16672659 PMC6674163

[B20] Satya N. S. Prakash D. V. S. Meena V. (2012). Purification of cinnamaldehyde from cinnamon species by column chromatography. Int. Res. J. Biol. Sci. 1, 49–51. Available online at: http://www.isca.in/IJBS/Archive/v1/i7/9.ISCA-IRJBS-2012-149.pdf

[B21] Smith C. A. Countryman R. A. Sahuque L. L. Colombo P. J. (2007). Time-courses of fos expression in rat hippocampus and neocortex following acquisition and recall of a socially transmitted food preference. Neurobiol. Learn. Mem. 88, 65–74. 10.1016/j.nlm.2007.03.001 17448703

[B22] Spence C. (2015). Multisensory flavor perception. Cell 161, 24–35. 10.1016/j.cell.2015.03.007 25815982

[B23] Squire L. R. Bayley P. J. (2007). The neuroscience of remote memory. Curr. Opin. Neurobiol. 17, 185–196. 10.1016/j.conb.2007.02.006 17336513 PMC2277361

[B24] Squire L. R. Genzel L. T. Wisted J. Morris R. G. M. (2015). Memory Consolidation. Cold Spring Harb. Perspect. Biol. 7:a021766. 10.1101/cshperspect.a021766 26238360 PMC4526749

[B25] Tayler K. K. Tanaka K. Z. Reijmers L. G. Wiltgen B. J. (2013). Reactivation of neural ensembles during the retrieval of recent and remote memory. Curr. Biol. 23, 99–106. 10.1016/j.cub.2012.11.019 23246402

[B26] Teixeira C. M. Pomedli S. R. Maei H. R. Kee N. Frankland P. W. (2006). Involvement of the anterior cingulate cortex in the expression of remote spatial memory. J. Neurosci. 26, 7555–7564. 10.1523/jneurosci.1068-06.2006 16855083 PMC6674278

[B27] Wrenn C. C. Harris A. P. Saavedra M. C. Crawley J. N. (2003). Social transmission of food preference in mice: methodology and application to galanin-overexpressing transgenic mice. Behav. Neurosci. 117, 21–31. 10.1037/0735-7044.117.1.21 12619904

[B28] Zovkic I. B. Paulukaitis B. S. Day J. J. Etikala D. M. Sweatt J. D. (2014). Histone H2A.Z subunit exchange controls consolidation of recent and remote memory. Nature 515, 582–586. 10.1038/nature13707 25219850 PMC4768489

